# A devastating complication from neonate knee septic arthritis due to conservative treatment: A case report

**DOI:** 10.1016/j.ijscr.2023.108790

**Published:** 2023-09-09

**Authors:** Hilmi Muhammad, Ardicho Irfantian

**Affiliations:** Pediatric Orthopaedic Subdivision, Orthopedic and Traumatology Division, Department of Surgery, Faculty of Medicine, Universitas Gadjah Mada, RSUP Dr. Sardjito Yogyakarta, Indonesia

**Keywords:** Neonate, Knee septic arthritis, Non-operative treatment, Complication, Case report

## Abstract

**Introduction:**

Septic arthritis in the neonate is a devastating condition that affects children and causes irreversible limb dysfunction or deformity. Neonatal septic arthritis is harmful and will end with skeletal abnormalities.

**Presentation of case:**

Neonate born with ileal atresia and underwent surgical treatment. Postoperatively, the patient experienced sepsis and was accompanied by septic arthritis. The patient was given triple IV antibiotic treatment without surgical debridement. Ten months later the deformity became prominent with physeal destruction of the affected area at distal femur. At age six the patient came to the orthopaedic outpatient clinic and there was a 3 cm limb-length discrepancy of both legs.

**Discussion:**

Early diagnosis of septic arthritis is critical for successful treatment, since neonates with delayed proper diagnosis have been shown to have poor long-term prognosis. In our case the patient was only managed by IV antibiotics administration and continued with oral antibiotics. The reason for this decision due to general condition was improved after medication although clinically he still has a small amount of knee swelling.

**Conclusion:**

Neonatal septic arthritis is dangerous and may have a devastating long term complication. Surgical management should be considered as treatment of choice if there is a lack or no progression from clinical and laboratory examination after antibiotic adiminstration. Growth arrest on the distal femur will result in leg length disparity and angular deformity.

## Highlights

• Septic arthritis in the neonate is a devastating condition that affects childrens and causes irreversible limb dysfunction or deformity.
• We present a case of the patients with neonatal septic arthritis treated only using IV antibiotics without surgical debridement.
• Surgical management should be considered as treatment of choice if there is a lack or no progression from clinical and laboratory examination after antibiotic administration.

## 1 Introduction

Septic arthritis in neonates is a devastating condition that affects many children and causes irreversible limb dysfunction or deformity. The most frequent presenting symptoms were erythema of the overlying skin, joint swelling, increased temperature, and increased white blood cell count [1]. Septic arthritis is positive if have 3 out of the following conditions: X-ray examination showing joint space widening or bone erosion; an increase in erythrocyte sedimentation rate, white blood cell count, and C-reactive protein; joint aspiration reveals pus in the articular space [2].

Not all septic arthritis will have positive pus culture as Heyworth et al. described that culture-negative septic arthritis if a synovial fluid WBC count of >50,000 WBCs/mm^3^ or >25,000 cells/mm^3^ with negative findings on synovial fluid microbiology culture [3]. CRP has subsequently been shown to be an excellent marker of septic arthritis and more useful than ESR.

Neonates are at risk from Gram-positive pathogens infection, like *S. aureus* and group B Streptococci, and Gram-negative organisms. For that reason, penicillin can be mixed with an aminoglycoside, such as gentamicin for Gram-negative coverage. The most common cause of SA in children older than one month is *S. aureus*, so empirical therapy should include coverage for *S. aureus* and may be administered following joint aspiration [3].

A delayed diagnosis resulted in severe complications, including more severe infections that require multiple irrigation and debridement, devastating cartilage destruction, femoral head osteonecrosis, and leg length discrepancy due to physeal bar destruction. This case report aimed to highlight the importance of early diagnosis and treatment of neonatal septic arthritis.

## 2 Case presentation

**Fig. 1:**
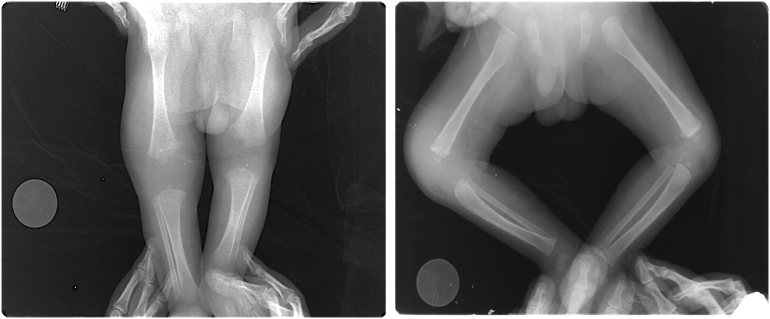
A 1-month-old boy radiograph X-ray of bilateral knee joint with septic arthritis of the right knee joint.

**Fig. 2:**
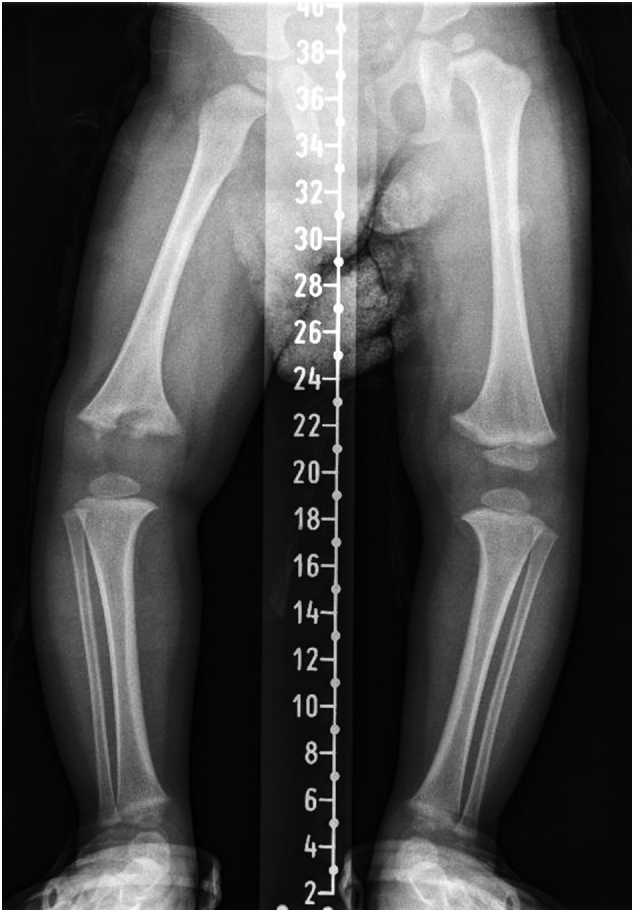
A 21-month-old boy radiograph long leg stitch view X-ray. The X-ray showed a destruction at the right distal femur growth plate.

This case is reported in line with the SCARE criteria [4]. The patient was born in February 2017 spontaneously with birth weight of 3000 g and sufficient gestational age with ileal atresia and underwent exploration laparotomy, Santulli Procedure, and ileostomy side-to-side by pediatric surgeon at Sardjito General Hospital a week later (Fig. 1). Previous antenatal care showed no abnormalities. There were no history of post illness within the family. Unfortunately, the patient experienced sepsis within post-operative period accompanied by septic arthritis on the right knee. The right knee was swollen, patients refused to move the right knee (Fig. 2). The X-ray was obtained the result was suggestive towards septic arthritis. It was confirmed by joint biopsy. Culture of biopsy shown a result of *Acinobacter baumanii*. On the other hand, *Serratia marcescens* and *Candida tropicalis* was identified by the blood culture result. Laboratory parameters shown an elevated level of CRP, Leucocyte and Procalcitonin suggestive to sepsis.

However, triple IV antibiotic treatment namely; Ampicillin sulbactam 100 mg/kg/day; divided every 12 h, Amikacin 15 mg/kg/BW/dose 24 hourly, and Metronidazole 7.5 mg/kg/dose 12 hourly, was given. The overall condition is not suitable for debridement. Attending physician considered to delay the debridement. However, the condition were improved. CRP level was significantly reduced from 146 to 37, with white blood count relatively normal range (8.04–8.57). After two weeks, the CRP was decreased to 6.0, and WBC was increased to 20. The clinical symptom of the knee is getting better, with a small amount of swelling, without surgical procedure given to the knee.

**Fig. 3:**
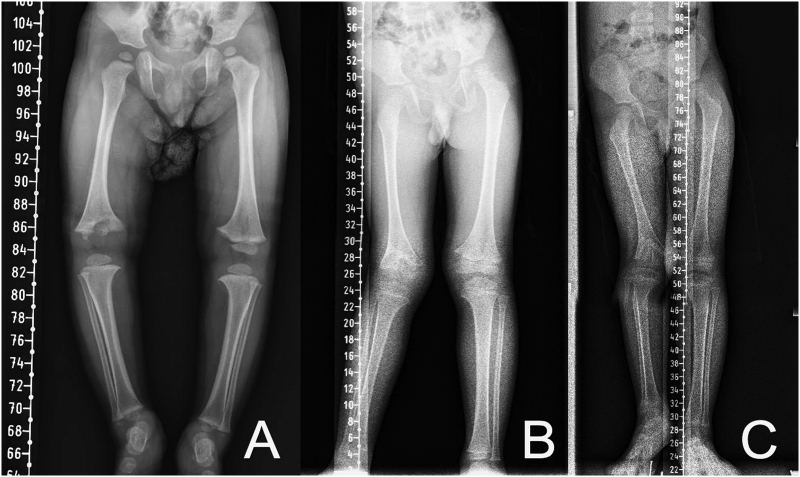
A. A 22-month-old boy radiograph long leg stitch view X-ray evaluation with O-shaped lower extremity. B. 10 months later the radiograph evaluation showed the lower extremity became X-shaped. C. 14 months later there was a 3 cm limb-length discrepancy.

At age two, the patient was again admitted to orthopaedic outpatient clinic. His parents complain about O-shaped legs, as seen on the X-ray (Fig. 3A). Ten months later the deformity became X-shaped, with physeal destruction of the affected area at the distal femur (Fig. 3B).

After several years, at age six, when the patient started school, he was again admitted to the orthopaedic outpatient clinic because of gait abnormality and the limb discrepancies of 3-cm.

## 3 Discussion

According to Obey (2019), early diagnosis of septic arthritis is critical for joint preservation since neonates with a delayed appropriate diagnosis have been shown to have a poor long-term prognosis [7]. Li Y et al. (2016) reported no significant difference between the surgical and nonsurgical treatment in neonates [2]. Frederiksen et al. (1993) also found that 66.7 % of infants with septic arthritis treated without surgery healed without squealing [6]. In our case, the patient was only managed by IV antibiotics administration and continued with oral antibiotics. The attending neonatology considered avoiding further investigations and early debridement since the patient had just undergone several procedures, and the overall condition was considered need to be improved first. However, the general condition improved after medication, although he still has a small amount of swelling. At age 6, we found physical growth plate destruction that caused a 3 cm limb-length discrepancy in both legs.

There is currently no consensus regarding the duration of intravenous therapy for newborns with septic arthritis. Some have suggested four to six weeks of intravenous antibiotics. Nonetheless, most research on osteomyelitis and septic arthritis in infants indicates shorter intravenous regimens (from approximately 1–4 weeks), but some authors recommend two-week intravenous antibiotics followed by two weeks of oral antibiotics [2,5]. However, surgical debridement should adjunct antibiotics therapy. Delayed debridement is allowed for 2 weeks, while after 2 weeks there are no difference seen in patients treated operatively or conservatively [14].

According to Vidigal (1998), avascular necrosis in infants and children with pyogenic arthritis has been attributed to either the compression of nutrient vessels by the increased intra-articular hydrostatic pressure caused by purulent exudate or to the septic vascular thrombosis caused by osteomyelitis [8]. Chondrolysis caused by septic thrombosis and the direct corrosive effect of purulent exudate in newborns [7]. Bacterial toxins and a cascade of cytokines from injured tissue trigger the acute-phase response. These enzymes from injured tissue and reacting leukocytes and bacterial toxins will cause articular cartilage degradation. The administration of antibiotic medication or surgical debridement can stop the ongoing activation of the acute phase response [9]. Distal femoral epiphysis consists of a physeal plate which is responsible for the growth of the majority of the femur and more than one-third of the overall length of the lower extremity, and it is the fastest-growing physis, with a growth rate of 1 cm/year. Growth arrest on the distal femur will result in angular deformity and leg length disparity [10]. Early diagnosis and accurate treatment of septic arthritis is the key to avoiding complications such as joint destruction, ankylosis, growth arrest, or the spread of infection leading to osteomyelitis or nerve lesions [11,12].

Growth arrest is one of the devastating complications that causes a leg length discrepancy. According to Dabash et al. (2018), the primary objectives of physeal growth arrest treatment are to correct deformity without resulting in additional aesthetic or functional deficits, restore limb length and mechanical alignment, and mobilize adjacent joints. The damaged physeal tissue cannot be repaired using any of the numerous surgical techniques currently available, including image-guided and arthroscopic physeal bar removal, chondrodiastasis, epiphysiodesis, and limb lengthening deformity correction, or a combination of these procedures [13].

A corrective osteotomy may be considered if the patient has a significant angular deformity. Whether or not to perform a corrective osteotomy with partial bridge resection must be based on the patient's remaining growth potential, the severity of the deformity, and the size of the physeal bridge. Epiphysiodesis may be used if the physeal bar is >50 % and excision is not possible due to the extent of the bony bridge. Chondrodiastasis, also known as physeal distraction, is another method for correcting deformities [12]. In our case, the patient still has >50 % of the physeal bar with a 3 cm leg length discrepancy without any comorbidity, thus planned for epiphysiodesis and corrective osteotomy to restore limb length and anatomical and mechanical alignment.

## 4 Conclusion

Neonate septic arthritis may have devastating long-term complications. Surgical management should be considered as the treatment of choice.

## Provenance and peer review

Not commissioned, externally peer-reviewed.

## Ethical approval

Ethical approval was waived for this case report by the XXX Ethics Committee. It was a retrospective case and no patient identity was disclosed nor a novel approach was applied.

## Parental consent for minors

Written informed consent was obtained from the patient's parents/legal guardian for publication and any accompanying images. A copy of the written consent is available for review by the Editor-in-Chief of this journal on request.

## Sources of funding

The authors declare that this study had no funding resources.

## CRediT authorship contribution statement

Muhammad, H: Conceptualization, Supervising, Editing, Writing.

Ardicho, I: Reviewing, Editing.

## Research registration

N/A.

## Guarantor

Hilmi Muhammad.

## Declaration of competing interest

The authors have no conflicts of interest to declare.
